# Supplementary material for: Aruncus dioicus var. kamtschaticus Extract Ameliorates Psoriasis-like Skin Inflammation via Akt/mTOR and JAK2/STAT3 Signaling Pathways in a Murine Model
Source: Nutrients. 2022 Dec 1;14(23):5094. doi: 10.3390/nu14235094 (PMC9736163; doi:10.3390/nu14235094)

**Supplementary material**

**Figure.S1.** The cytotoxic effect of ADE on cell viability of HaCaT cells. Cells were treated wit h multiple-dose of ADE for 24 hours and cell viability was measured by MTT assay. The data are shown as the means ± standard deviation. ADE: *A.dioicus* extract.

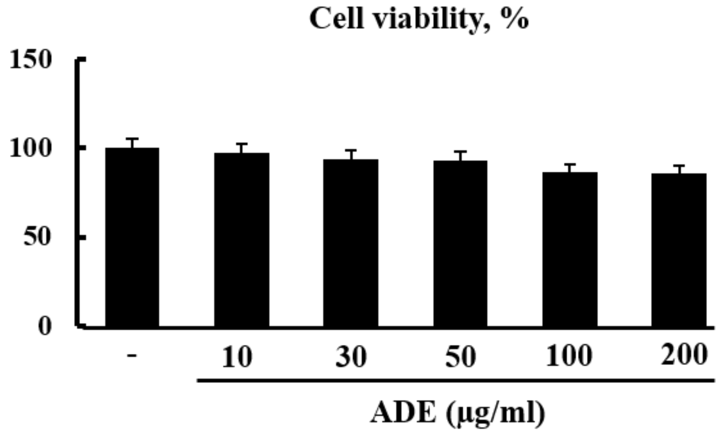

Supplement: Supplementary file 1 [file nutrients-14-05094-s001.zip › nutrients-2017152-supplementary.pdf]
